# Effects of Sucrose Palmitate on the Physico-Chemical and Mucoadhesive Properties of Buccal Films

**DOI:** 10.3390/molecules25225248

**Published:** 2020-11-11

**Authors:** András Kelemen, Bálint Katona, Szilvia Módra, Zoltán Aigner, István Sebe, Klára Pintye-Hódi, Romána Zelkó, Géza Regdon, Katalin Kristó

**Affiliations:** 1 Department of Applied Informatics, University of Szeged, Boldogasszony sgt. 6., H-6725 Szeged, Hungary; kelemen@jgypk.u-szeged.hu; 2 Institute of Pharmaceutical Technology and Regulatory Affairs, University of Szeged, Eötvös u. 6., H-6720 Szeged, Hungary; balint.katona@bitrial.hu (B.K.); modra.szilvia@gmail.com (S.M.); aigner@pharm.u-szeged.hu (Z.A.); klara.hodi@pharm.u-szeged.hu (K.P.-H.); kristo@pharm.u-szeged.hu (K.K.); 3 University Pharmacy Department of Pharmacy Administration, Semmelweis University, Hőgyes E. u. 7-9., H-1092 Budapest, Hungary; istvan.sebe@gmail.com (I.S.); zelko.romana@pharma.semmelweis-univ.hu (R.Z.)

**Keywords:** mucoadhesion, buccal film, mucoadhesive work, sucrose-palmitate

## Abstract

In our current research, sucrose palmitate (SP) was applied as a possible permeation enhancer for buccal use. This route of administration is a novelty as there is no literature on the use of SP in buccal mucoadhesive films. Films containing SP were prepared at different temperatures, with different concentrations of SP and different lengths of hydroxypropyl methylcellulose (HPMC) chains. The mechanical, structural, and in vitro mucoadhesive properties of films containing SP were investigated. Tensile strength and mucoadhesive force were measured with a device and software developed in our Institute. Positron annihilation lifetime spectroscopy (PALS) and X-ray powder diffractometry (XRPD) were applied for the structure analysis of the films. Mucoadhesive work was calculated in two ways: from the measured contact angle and compared with direct mucoadhesive work, which measured mucoadhesive force, which is direct mucoadhesion work. These results correlate linearly with a correlation coefficient of 0.98. It is also novel because it is a new method for the determination of mucoadhesive work.

## 1. Introduction

Nowadays, bioadhesive formulations are increasingly becoming a very important class of dosage in the forms of films [1,2,3], tablets, nanoparticles [4,5], etc. The buccal administration of active ingredients by mucoadhesive films is one of the most innovative ways that can be used to achieve systemic drug delivery. Because of their ease of use, they have good patient compliance and can be used in elderly people, children [6], or patients with difficulty in swallowing. The great advantage of this administration route is that the active ingredient is introduced into the bloodstream without degradation and avoids the first-pass effect. The development of buccal mucoadhesion formulations is, therefore, a huge exploitable area in pharmaceutical technology.

The basis of buccal mucoadhesive preparations are polymers with mucoadhesive properties, such as hydroxypropyl-methylcellulose (HPMC), poly-acrylic acid copolymer [7], chitosan [8], or sodium hyaluronate [9]. Plasticizer additives can be used in the film to make the administration easier by increasing the elasticity of the preparations, for instance, glycerine or polyethylene glycol [10]. During the formulation of buccal mucoadhesive films, some excipients can be applied (e.g., ethanol, taste masking agent, permeation enhancers, etc.) [11].

There is a great number of film-forming agents for the preparation of buccal mucoadhesive films. Preis et al. (2014) formulated and investigated two-layer films containing lidocaine. Aqueous solutions of hydroxypropyl methylcellulose (HPMC), methylcellulose (MC), PEG-polyvinyl alcohol copolymer (PPACP), and hydroxyethyl cellulose (HEC) were used for the formulation of the adhesive layer during film-forming. The aim of this experiment was to investigate the effect of materials and preparation methods on the properties of this form of dosage, such as disintegration time, dissolution profile, and the structure of the polymer film. The quality of materials that can be used for the protecting layer was optimized in this film [9].

Cavallari et al. (2015) developed buccal mucoadhesive films containing a natural gel-forming polymer from Psyllium and sodium carboxymethyl cellulose (SCMC). These cast films were compared with HPMC films. Film casting was performed from an aqueous solution. The active agent was dissolved, and permeation and microbiological tests were performed. The result showed no significant difference between the parameters of the SCMC copolymer containing Psyllium film and the HPMC film [12].

In the literature, there are a lot of active agents that are applied in buccal mucoadhesive films. Active agents with both systemic and local effects can be applied in these films. Shiledar et al. (2014) formulated two-layer films containing zolmitriptan as an active agent in xanthan gum (XG) and a HPMC base. The aim of this work was to study the behavior of XG and zolmitriptan in this form of dosage. XG met the requirements as a film-forming agent and also as bioadhesive material. The penetration of zolmitriptan through the buccal membrane was narrow; therefore, the application of a permeation enhancer excipient is necessary for this form of dosage [13].

Mura et al. (2015) prepared cross-linked wafers containing econazole nitrate from the aqueous solution of methoxy amidated pectin and carboxymethyl cellulose (CMC) with the application of a freeze-drying technique. Econazole nitrate has an antimycotic effect; thus, this dosage form was developed for treating oral candidiasis. It can be concluded LMAP-CMC buccal mucoadhesive wafer containing econazole nitrate has an antimycotic effect in this form of dosage [14].

During the formulation of buccal mucoadhesive films, some excipients can be applied as a permeation enhancer, taste masking agent, a protective layer polymer, and plasticizer. Mašek et al. (2017) performed experiment work related to vaccination through the buccal and sublingual mucus. For this work, a complex three-excipient layered system was needed. Bioadhesion was ensured by one mucoadhesion layer, which was a film cast from an aqueous solution of Carbopol:HPMC 2:1 ratio. Methacrylic acid in ethyl alcohol solution was used as a protecting layer. The innermost layer was a nanofiber system made up of three different reservoirs. Sodium deoxycholate was used as a permeation enhancer. Based on the results, buccal mucoadhesive films containing nano-vehicles were found suitable for further investigation and for the introduction of an immunologically active substance into the reservoir [15].

Sucrose esters are synthesized by the esterification of fatty acids with sucrose [16]. In our current research, sucrose-palmitate (SP) was assayed as a possible permeation enhancer for buccal use, which would be an absolutely innovative additive for this route of administration. Films containing SP were prepared, and their mechanical, structural, and in vitro mucoadhesive properties were investigated.

## 2. Results and Discussion

### 2.1. Tensile Strength and Deformation Process

**Figure 1:**
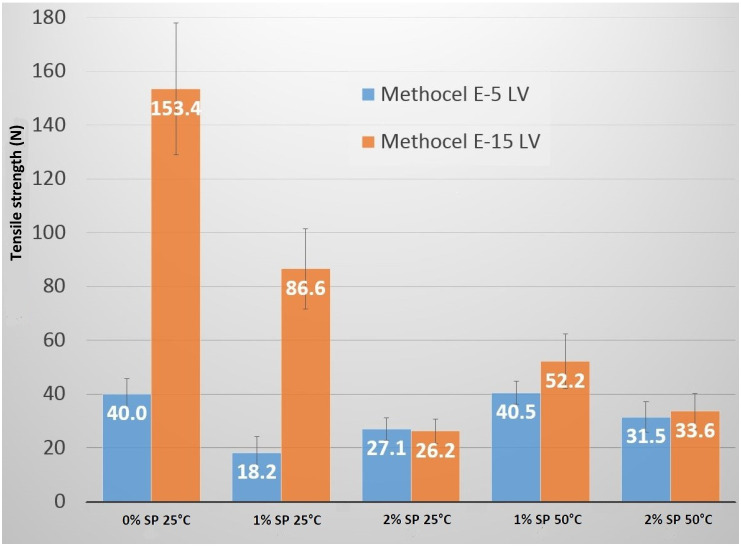
The results of tensile strength (blue: HPMC-5 (Methocel E5-LV); orange: HPMC-15 (Methocel E15-LV)). HPMC: hydroxypropyl methylcellulose.

The type of HPMC affected the tensile strengths of the films. The films that were formed using 15 cp HPMC always showed higher values than the ones formed using 5 cp because the average molecular weight of HPMC is higher; therefore, the films have better mechanical properties (Figure 1). In the case of 2% SP concentration, tensile strength was independent of the polymer type because this amount could not be incorporated in the polymer structure entirely; only a lower amount in the case of both polymers could be incorporated. It could be correlated with the XRPD results because when using 2% SP, the characteristic peaks of SP were higher than in the case of 1%.

The tensile strength results of the films were compared to obtain information about the effect of the different amounts of the additive used (Figure 1). The tensile strengths were analyzed for the films made of the same type of HPMC, at the same temperature, but with different SP concentrations related. All the films containing SP had lower tensile strength values than the films with HPMC only. The increase in the concentration of SP from 1% to 2% in the casting solution resulted in lower measured tensile strength values, but this decrease was not significant. HPMC-5 at 25 °C was an exception as tensile strength increased with the increasing concentration of SP. This can be explained by the fact that more SP molecules can be incorporated into a shorter polymer chain than into a longer one without changes in the mechanical properties.

The effect of temperature on the measured tensile strength values was also analyzed. The films compared had the same concentration of HPMC and SP but different solving, casting, and drying temperatures. It can be seen that the films prepared at 50 °C manufacturing temperature had a higher tensile strength than the films prepared at 25 °C. The type of HPMC did not affect this phenomenon. In the case of 2% SP, the temperature did not cause any change in tensile strength.

**Figure 2:**
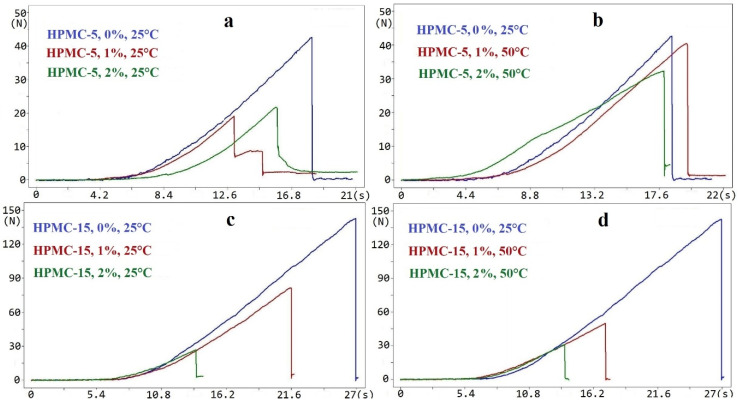
Deformation process in the case of HPMC and HPMC films containing SP. (**a**) HPMC-5, 25 °C; (**b**) HPMC-5, 50 °C; (**c**) HPMC-15, 25 °C; (**d**) HPMC-15, 50 °C). SP: sucrose-palmitate.

The deformation curves of the deformation process were also analyzed. On the curves of the deformation process, there was a breaking point where the force was the maximum value, after which it decreased to zero (Figure 2). It correlates with our previous results [17]. In the case of 50 °C temperature and the application of SP, the curves were more stretched because the films had a more elastic deformation. It can be seen that SP showed a plasticizer effect in this composition.

### 2.2. Surface Free Energy

The surface free energy (SFE) and the polar and disperse components of the ingredients and the films were calculated from the measured contact angle (Table 1 and Table 2). The dispersed part was from the London dispersion force, while the polar part was from dipole-dipole interaction, induction force, and H-bonds. The investigation of the ingredients revealed that SP had the highest polar and total surface free energy. HPMC-5 and HPMC-15 powders had similar wetting properties.

The SFE values of HPMC powder and HPMC films were different. The polar and the total parts increased in the case of films, while the disperse component decreased because the dipole-dipole interaction, induction force, and H-bonds became stronger in the films than in the powder form. H-bonds can also form at the end of polymer chains because OH groups are also found there. Therefore, it was hypothesized that the shorter polymer chain might form slightly more H-bonds, which may result in a higher SFT because the same weight% was used for both films.

When SP was applied, the disperse component of the SFE of the films was lower, while the polar part, as well as the total SFE, were higher than in films without SP. The application of SP can indicate more H-bonds between HPMC and SP molecules; therefore, the polar part became higher. Increasing the amount of the SP added caused an increase in the total SFE in every case. When HPMC-5 polymer was used, the polar component was higher with 1% SP than with 2% SP because 2% SP could not be incorporated in the polymer chain; therefore, the disperse parts were increased because of the London dispersion force. In the case of HPMC-15 polymer films with 1% SP, the polar part was lower than with 2% SP because the polymer chain was longer, and fewer H-bonds could be generated.

The melting point of SP is 48 °C; therefore, it melts at a temperature of 50 °C. It can be assumed that it is able to form more chemical bonds in the system, which can lead to higher SFE values. This phenomenon was clearly observed at 2% SP concentration for both polymers, as higher SFE values could be measured for films obtained at 50 °C than for films obtained at 25 °C. However, at a concentration of 1% SP, there was either no change in SFE or only a minimal increase. This may be due to the fact that at a concentration of 1%, SP did not cause a measurable difference under the influence of temperature.

### 2.3. Spreading Coefficient

The contact angle was measured on the surface of the comprimates made from pure starting materials. First, the total and the polar and disperse parts of surface free energy were calculated (Table 3) from these data. The polar component of SP was high, which can be explained by the fact that the hydrophilic–lipophilic balance (HLB) value of SP was 16, which is typical of a more hydrophilic material.

The spreading coefficients were calculated according to Equation (2) from these data (Table 3). It can be seen that HPMC spread over the surface of SP, which means that the properties of HPMC will dominate on the surface of the films obtained.

### 2.4. Mucoadhesive Force

Based on literature data, HPMC has very good mucoadhesive properties and therefore is excellent for the formulation of buccal mucoadhesive films [18,19,20], which was also supported by our results.

**Figure 3:**
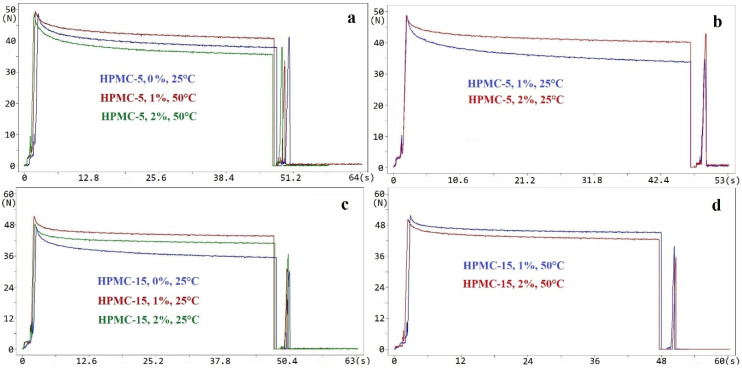
In vitro mucoadhesive force of HPMC and HPMC containing SE films. (**a**) HPMC-5, 50 °C; (**b**) HPMC-5, 25 °C; (**c**) HPMC-15, 25 °C; (**d**) HPMC-15, 50 °C.

We also studied the course of the curves obtained when measuring the mucoadhesion of the various samples (Figure 3). There was no significant difference between the characteristics of the curves. Neither the concentration nor the manufacturing temperature affected the characteristics.

The force values needed to tear apart the surfaces after diffusion were compared (Table 4). It was studied how the different film-forming polymers, compositions, and casting temperatures affected the mucoadhesive force of the films. The films made from polymers of different molecular weights and with the same polymer/SP ratio and casting temperature were compared, and a significantly lower level of mucin diffusion force was found in the films made with HPMC-15. There can be two reasons for this. The larger molecules move more slowly in passive diffusion (Fick’s first law), and in this way, the 15 cp films would have lower diffusion force, making it more difficult for the mucin molecules to penetrate into them. Using the same polymer and temperature but a different additive ratio during casting had an effect on mucoadhesive force. The films without SP had better mucin adhesive properties in all the cases; however, SP did not reduce them significantly because it contains OH groups that are also capable of forming H-bonds with mucin. There were differences between the films containing different SP concentrations. In the case of a higher SP concentration, the mucoadhesive force was higher. The reason can be that the 2% SP content has many OH groups, but at this concentration, the total amount of SP could not be incorporated into the polymer structure, so free SP molecules remained in the system, resulting in more free OH groups than the incorporated molecules. These free OH groups could form more H-bonds with mucin. This phenomenon may explain why the higher SP content resulted in higher mucoadhesive force. A relationship was found between mucoadhesive force and tensile strength. Where the mucoadhesive force was higher, the tensile strength was also higher, with the exception of 2% SP containing films because 2% SP was too high an amount, the entire amount could not be incorporated in the polymer structure.

Each time the temperature was higher during manufacturing, higher values of mucoadhesive force were obtained. The only exception was the films prepared at 50 °C with 1% SP and HPMC-15. In this case, a lower mucoadhesive force was measured than at 25 °C. To gain information about the reasons behind the difference between the mucoadhesive force values of the films with and without SP, and between the films made at 25 °C and 50 °C, the surface properties of the films were studied, and the surface free energy of the films was calculated.

**Figure 4:**
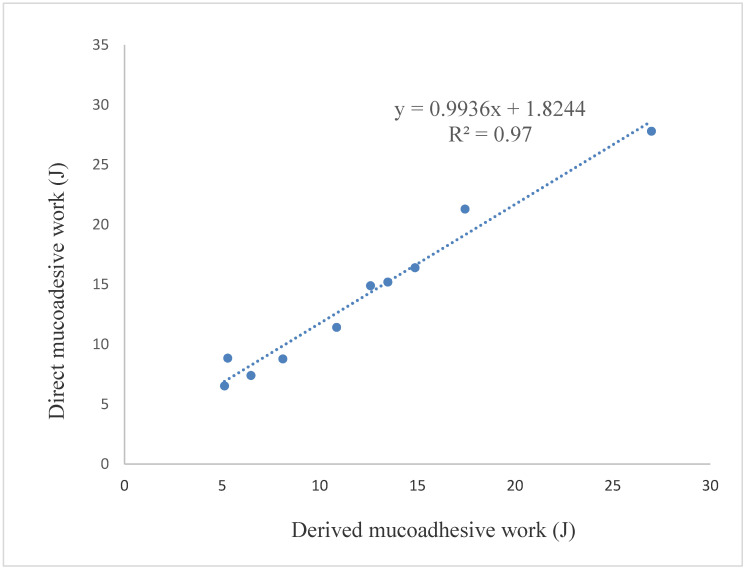
Correlation between the direct and the derived mucoadhesive work.

Mucoadhesive work was calculated from the measured mucoadhesive force with the equation W = F · s, where W denotes the work, F denotes the force, and s denotes the displacement. This value is the direct mucoadhesive work in Figure 4. Mucoadhesive work was also calculated from the results of the measured contact angle. In this case, the Young–Dupree equation was used. It can be seen that the correlation between the two values of mucoadhesive work was linear with a correlation coefficient of 0.98. Therefore mucoadhesive work can be predicted from the measured contact angle, and according to our results, these data are in good correlation with the mucoadhesive work calculated from the measured mucoadhesive force.

First, the primary physical and in vitro mucoadhesive properties of the films were examined, and then the structure of the films was analyzed to obtain information about the reasons behind the previously mentioned properties.

### 2.5. X-ray Powder Diffraction (XRPD)

**Figure 5:**
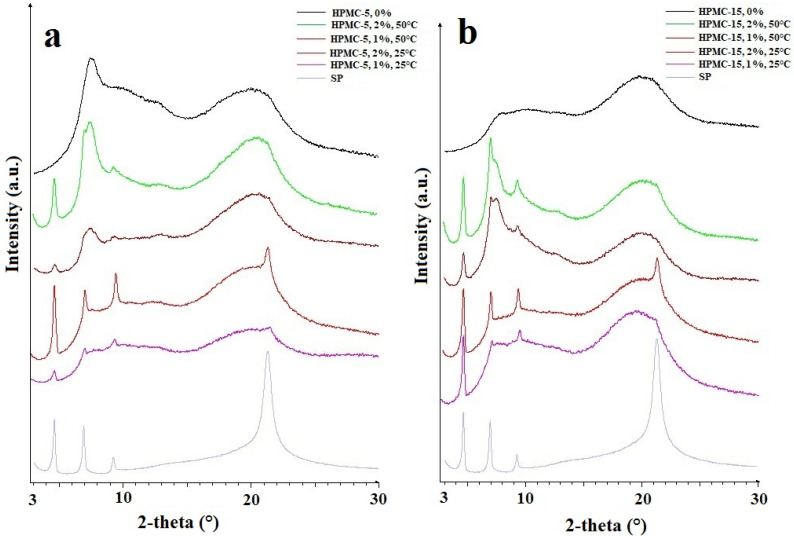
XRPD spectra of HPMC-5 and HPMC-15 films. (**a**) HPMC-5; (**b**) HPMC-15.

The components of films, HPMC and SP, were measured as a powder form with X-ray powder diffractometry (XRPD). HPMC is an amorphous material, while SP is a semi-crystalline material and had four characteristic peaks (4.59, 6.90, 9.23, and 21.31 2-theta) (Figure 5). These results were in agreement with the literature data [21]. The X-ray spectra of the films were compared with the starting materials, and the net area was calculated for the first peak (4.59 2-theta) (Table 5) because, in this case, there was a well-defined baseline. The net area was found to be lower for the films obtained at 50 °C than for the films obtained at 25 °C, which means a lower crystal grade. It can be seen that more SP was incorporated in the polymer structure at 50 °C than at 25 °C because the crystal grade was lower when the same concentration of SP was used. For HPMC-15 this HPMC peak at 50 °C was lower, and the second peak (6.9 2-theta) of SP was higher than for HPMC-5. It can be concluded that in this case, a greater amount of SP was incorporated into the HPMC-5 polymer structure than into HPMC-15. The reason for this can be that SP can be incorporated in between the polymer chains, therefore in the case of longer polymer chains, this amount is lower. The tensile strength results also suggest this because in the case of HPMC-15 and 1% SP containing films, the tensile strength decreased from 153.4 N to 52.2 N, but in the case of HPMC-5 there was no significant difference (40 N and 40.5 N).

### 2.6. Positron Annihilation Lifetime Spectroscopy (PALS)

Positron annihilation lifetime spectroscopy (PALS) was performed to obtain information about the free volumes within the films. The films with and without SP were measured. As a reference, HPMC-5 and HPMC-15 free films were used. The lifetime of ortho-positronium (o-Ps) was between 1924 and 1988 ps. There was no significant difference between the results of the films. It can be concluded that the o-Ps lifetime values were not influenced by the temperature, the concentration of SP or the polymer type either, which indicates that the average free volume holes of different samples were similar. These films are potentially applicable as carrier films; therefore, the PALS results provide valuable information about the supramolecular distribution of the active agent selected for further application. These results are very similar to our previous results where we studied hydroxypropyl cellulose (Klucel) films, and the results were between 1860–2000 ps [22].

## 3. Materials and Methods

### 3.1. Materials

Methocel E-5 LV (HPMC-5) and E-15 LV (HPMC-15) (Merck Co. Ltd., Darmstadt, Germany) were used as film-forming polymers in the concentrations of 6.5, 7.5, and 8.5 *w*/*w*%. The viscosity of (HPMC-5) and Methocel E-15 LV is 5 cp and 15 cp, respectively. These data were supplied by the producer. Ryoto^®^ P1670 (Mitsubishi-Kagaku Foods Corporation, Tokyo, Japan) sucrose palmitate (SP) (sugar ester) was added to the polymer solution in the concentration of 1 and 2 *w*/*w*%. The HLB value of SP is 16, and its melting point is 48 °C (data by Mitsubishi-Kagaku Foods Corporation). Distilled water was used for polymer solutions as a solvent.

### 3.2. Methods

#### 3.2.1. Preparation of Samples

The solvent casting method was used for the preparation of the films on the Teflon surface. This method is widespread and easy to apply to the production of buccal films [23]. Two different temperatures were used for the preparation of the solutions and drying: 25 °C and 50 °C, 65% relative humidity (RH) over 24 h.

For each type of HPMC, five samples different with respect to the HPMC and SP content, and the temperature of preparation were obtained (Table 6). The content of dry material was standard in all the samples.

#### 3.2.2. Tensile Strength

Tensile strength was tested with a device and software developed in our Institute; the in vitro mucoadhesive force of the film on mucin solution can be measured as well [24]. This device contains a special holder (20 mm in diameter) and a hemispherical indent with a surface area of 201 mm^2^ and is connected to a computer through an interface. The final deformation force can be measured, and the deformation process (force-time and force-displacement curves) can be followed. The circular holder is situated horizontally, and the jowl moves vertically. The measuring range was 0–200 N, the speed of the stamp was 20 mm/min, the sampling rate was 50 Hz, the output was 0–5 V, and the sensitivity was ±0.1. The sensor comprised UNICELL force-measuring equipment, calibrated with the C9B 200 N cell; 10 parallel measurements were performed on each specimen.

#### 3.2.3. Surface Free Energy (SFE)

The calculation of the surface free energies (SFE) of the films was based on the results from contact angle measurements with OCA 20 (DataPhysics Instruments GmbH, Filderstadt, Germany). This indirect method of assessing the surface free energy from wettability measurements is widely used [25,26,27]. In the method of Wu [28], surface free energy is taken as the sum of disperse (γ^d^) and polar (γ^p^) components. The surface free energy of solid materials can be determined by means of contact angle measurements with two different liquids with known polar and disperse parts of surface tension properties (Equation (1)). The test fluids were distilled water and diiodomethane (Merck KGaA, Darmstadt, Germany). According to Ström [29], the disperse part of surface tension was 21.8 mN/m for water and 50.8 mN/m for diiodomethane, while the polar part of surface tension was 51 mN/m for water and 0 mN/m for diiodomethane. Compacts of 0.50 g of powders were made with a hydraulic press (Specac Inc, Graseby, Orpington, UK), with a dwell time of 10 s, at a pressure of 200 MPa. Circle fitting was applied to determine the contact angle formed on the comprimates prepared from different samples. They can be assessed by solving an equation with two unknowns (Equation (1)):

(1) $$\left(1+\cos\theta \right)\gamma_{l}=\frac{4\left(\gamma_{s}^{d}\gamma_{l}^{d}\right)}{\gamma_{s}^{d}+\gamma_{l}^{d}}+\frac{4\left(\gamma_{s}^{p}\gamma_{l}^{p}\right)}{\gamma_{s}^{p}+\gamma_{l}^{p}}$$

where θ is the contact angle, γ_s_ is the solid surface free energy, and γ_l_ is the liquid surface tension (superscripts refer to their polar (γ^p^) and disperse part (γ^d^)).

#### 3.2.4. Spreading Coefficient

If the surface free energy of the solid materials is known, the spreading coefficient (S) may be computed, and the interactions between the two materials may be predicted (Equation (2)). The spreading coefficient is calculated as the difference between the adhesion work and the cohesion work. The two materials which interact can be two powders or a powder and a liquid. The spreading coefficient (S_12_) of a material (1) over the surface of another material (2) can be determined as follows [30] (Equation (2)):

(2) $$S_{12}=\frac{4(\gamma_{1}^{d}\gamma_{2}^{d})}{\gamma_{1}^{d}+\gamma_{2}^{d}}+\frac{4(\gamma_{1}^{p}\gamma_{2}^{p})}{\gamma_{1}^{p}+\gamma_{2}^{p}}-\frac{\gamma_{1}}{\gamma_{2}}$$

#### 3.2.5. Mucoadhesive Force and Mucoadhesive Work

Before in vitro adhesion strength experiments, the film thickness was measured with a bolt micrometer with an accuracy of 0.001 mm (Mitutoyo, Kawasaki, Japan). Mucin gel was in situ prepared before the study: 500 mg of mucin was mixed with 5 mL of distilled water. Fresh mucin gel was smeared to the bottom probe from measurement to measurement. Samples were measured at room temperature (20 ± 5 °C). The structure of the measurement system was as follows, from top to bottom: a stainless steel holder, a bilayer adhesive tape, a film, mucin gel, and a stainless steel table. Each element was measured alone, in pairs, and all together. First, the characteristics of the average curves of the mucin diffusion force measurements were analyzed. The process and curve can be divided into three phases. First, the instrument pushes the film, which is attached to the tool, toward the mucin solution on the bench with 50 N. In the second phase, the instrument keeps the position of the tool and the film for 45 s. During this time, the diffusion of the polymers into the surface and the mucin molecules into the film takes place. The slow decrease of the pressure strength could be seen on the curve. In this phase, the instrument maintains the position of the tool and the film and monitors the strength needed for doing so. As the film gets slightly compressed between the bench and the tool, the structure relaxes, making it easier for the instrument to maintain the position. In the third phase, the instrument starts to pull the tool. To maintain the movement speed, it needs to increase the pulling strength to the level of the mucin diffusion force, which keeps the film and the mucin stuck. This is a high peek during a short period on the curve. The highest value of the peek, where the instrument matches the mucin adhesion force, is the “in vitro mucin diffusion force”. Before the mucoadhesion test, each film was subjected to 50 N, which was held for 45 s, and the holder then pulled the film up from the mucin gel layer. At least ten parallel measurements were performed on each specimen.

The tensile strength tester was improved for the direct measurement of adhesion work, as described by Kelemen et al. [24]. Mucoadhesive work was calculated from the measured mucoadhesive force with (Equation (3))

(3) W=Fs

where W denotes the work, F denotes the force, and s denotes the displacement.

The indirect calculation of mucoadhesive work was performed on the basis of the Young-Dupré equation (wetting theory) (Equation (4)).

(4) $$W_{a}=\gamma_{l}\left(1+\cos\left(\theta \right)\right)$$

where W_a_, $\gamma_{l}$, and θ denote the work of adhesion, the surface tension of water (72.8 mN/m), and the contact angle, respectively [31,32,33].

#### 3.2.6. X-ray Powder Diffractometry (XRPD)

X-ray powder diffractometry (XRPD) can be applied for the investigation of the structure of films [34,35,36]. XRPD analysis was performed with a Bruker D8 Advance diffractometer (Bruker AXS GmbH, Karlsruhe, Germany) system with Cu K λI radiation (λ = 1.5406 Å). The samples were scanned at 40 kV and 40 mA from 3 deg to 40 deg 2θ, at a scanning speed of 0.1 deg/s and a step size of 0.010 deg. The amorphous/crystalline state of the tested polymer system and the possible recrystallization tendency with accelerated aging were examined.

#### 3.2.7. Positron Annihilation Lifetime Spectroscopy (PALS)

The application of PALS is an innovative instrumental process during structural analysis [37]. Gottnek et al. applied PALS for detecting the location of lidocaine hydrochloride as an active pharmaceutical ingredient in hydroxypropyl cellulose (HPC) films. This study also confirmed the applicability of PALS for the investigation of the microstructure of polymer films [22]. The lifetime spectrometer applied was constructed from BaF_2_ based detectors and standard ORTEC electronics. Spectra were collected in the 4096 channels of a multichannel analyzer. The time/channel value was ~10 ps and the time resolution of the system was ~210 ps. As a positron source, carrier-free ^22^NaCl was used, sealed between Kapton foils. The activity of the source was 10^6^ Bq, and only 5–8% of the positrons were annihilated in the source itself.

## 4. Conclusions

Based on our results, the polymer with a shorter chain can be recommended for further development. The mucin diffusion values were higher when the lower molecular weight HPMC was applied. SP did not have a significant effect on the suitable mucoadhesion properties of HPMC.

The potential permeation enhancer SP seemed to have plasticizer properties also when casting at room temperature, which is beneficial for the patient and profitable for the manufacturer. SP can be incorporated into the HPMC polymer structure, which was proven with XRPD analysis. SP can also behave as a plasticizer; therefore, it is a promising excipient in mucoadhesive buccal films. At a 1% concentration, it could be incorporated into the polymer structure, while at a 2% concentration, there were some free SP molecules in the films, so the application of a 1% SP can be recommended. SP can be recommended for use as a suitable additive, as a possible permeation enhancer, and a plasticizer for buccal mucoadhesive films. It could be determined that HPMC spread over the surface of SP, which means that the properties of HPMC would dominate on the surface of the films obtained. Neither the temperature nor the concentration of SP had an effect on the free volumes; therefore, all the films could be used for buccal administration.

Mucoadhesive work was calculated from the measured contact angle. It was compared with direct mucoadhesive work, which was calculated from the measured mucoadhesive force and displacement. These results were correlated, and a linear curve could be fitted with a correlation coefficient of 0.98. This study was the first to describe the relationship between the indirect mucoadhesive work calculated from SFE and the mucoadhesive work calculated from mucoadhesive force measured directly. The latter enables the novel determination of mucoadhesive work.

## Figures and Tables

**Table 1:** The results of surface free energy (**γ^tot^**) and polar (**γ^p^**) and disperse (**γ^d^**) components.

Sample	γ^tot^ (mN/m)	γ^d^ (mN/m)	γ^p^ (mN/m)
HPMC-5-0%-25 °C	64.40	38.01	26.39
HPMC-5-1%-25 °C	67.18	30.30	36.87
HPMC-5-2%-25 °C	67.68	37.08	30.60
HPMC-5-1%-50 °C	68.50	30.13	38.36
HPMC-5-2%-50 °C	70.54	32.08	38.46
HPMC-15-0%-25 °C	55.74	37.22	18.52
HPMC-15-1%-25 °C	65.59	35.35	30.24
HPMC-15-2%-25 °C	67.07	34.28	32.79
HPMC-15-1%-50 °C	64.79	35.84	28.95
HPMC-15-2%-50 °C	70.16	30.98	39.18

**Table 2:** The surface free energy results of starting materials.

Material	γ^tot^ (mN/m)	γ^d^ (mN/m)	γ^p^ (mN/m)
HPMC	55.19 ± 2.50	39.40 ± 2.02	15.79 ± 1.47
SP	71.11 ± 2.33	27.01 ± 1.50	44.10 ± 1.79

**Table 3:** Spreading coefficient of SP over HPMC (S_12_) and of HPMC over SP (S_21_).

Material 1	Material 2	S_12_ (mN/m)	S_21_ (mN/m)
SP	HPMC	−7.91	0.09

**Table 4:** The results of mucoadhesive force measurement. (*: reference value).

Sample	Mucoadhesive Force (N)	Standard Deviation (N)	Significant Difference (*p* < 0.05)
HPMC-5-0%	12.58	0.94	*
HPMC-5-1%-25 °C	9.96	2.00	No
HPMC-5-2%-25 °C	11.2	0.48	No
HPMC-5-1%-50 °C	10.35	1.39	No
HPMC-5-2%-50 °C	11.34	1.81	No
HPMC-15-0%	9.81	1.19	*
HPMC-15-1%-25 °C	8.41	1.37	No
HPMC-15-2%-25 °C	8.38	0.99	No
HPMC-15-1%-50 °C	7.82	0.68	No
HPMC-15-2%-50 °C	9.66	0.39	No

**Table 5:** The results of the X-ray powder diffractometry (XRPD) analysis.

Sample Name	Net Area
SP Powder	13.42
HPMC-5-1%-25 °C	0.821
HPMC-5-2%-25 °C	5.855
HPMC-5-1%-50 °C	0.763
HPMC-5-2%-50 °C	4.309
HPMC-15-1%-25 °C	4.72
HPMC-15-2%-25 °C	5.27
HPMC-15-1%-50 °C	2.13
HPMC-15-2%-50 °C	4.975

**Table 6:** The composition of the solutions for film casting.

Sample Name	HPMC (*w*/*w*%)	SP (*w*/*w*%)	Temperature (°C)
HPMC-5-0%	8.5	0	25
HPMC-5-1%-25 °C	7.5	1	25
HPMC-5-2%-25 °C	6.5	2	25
HPMC-5-1%-50 °C	7.5	1	50
HPMC-5-2%-50 °C	6.5	2	50
HPMC-15-0%	8.5	0	25
HPMC-15-1%-25 °C	7.5	1	25
HPMC-15-2%-25 °C	6.5	2	25
HPMC-15-1%-50 °C	7.5	1	50
HPMC-15-2%-50 °C	6.5	2	50
